# Same but different: Comparison of two system-specific molecular chaperones for the maturation of formate dehydrogenases

**DOI:** 10.1371/journal.pone.0201935

**Published:** 2018-11-16

**Authors:** Nadine Schwanhold, Chantal Iobbi-Nivol, Angelika Lehmann, Silke Leimkühler

**Affiliations:** 1 Institute of Biochemistry and Biology, Department of Molecular Enzymology, University of Potsdam, Potsdam, Germany; 2 Aix-Marseille Université, CNRS, BIP UMR7281, Marseille, France; University of Alberta, CANADA

## Abstract

The maturation of bacterial molybdoenzymes is a complex process leading to the insertion of the bulky bis-molybdopterin guanine dinucleotide (bis-MGD) cofactor into the apo-enzyme. Most molybdoenzymes were shown to contain a specific chaperone for the insertion of the bis-MGD cofactor. Formate dehydrogenases (FDH) together with their molecular chaperone partner seem to display an exception to this specificity rule, since the chaperone FdhD has been proven to be involved in the maturation of all three FDH enzymes present in *Escherichia coli*. Multiple roles have been suggested for FdhD-like chaperones in the past, including the involvement in a sulfur transfer reaction from the l-cysteine desulfurase IscS to bis-MGD by the action of two cysteine residues present in a conserved CXXC motif of the chaperones. However, in this study we show by phylogenetic analyses that the CXXC motif is not conserved among FdhD-like chaperones. We compared in detail the FdhD-like homologues from *Rhodobacter capsulatus* and *E*. *coli* and show that their roles in the maturation of FDH enzymes from different subgroups can be exchanged. We reveal that bis-MGD-binding is a common characteristic of FdhD-like proteins and that the cofactor is bound with a sulfido-ligand at the molybdenum atom to the chaperone. Generally, we reveal that the cysteine residues in the motif CXXC of the chaperone are not essential for the production of active FDH enzymes.

## Introduction

Molybdoenzymes comprise a large group of redox enzymes present in all kingdom of life [1]. Generally, molybdoenzymes are classified into three different families according to the ligands present at the molybdenum atom [2], namely the xanthine oxidase (XO) family, the sulfite oxidase (SO) family and dimethyl sulfoxide (DMSO) reductase family. While enzymes of the XO and SO families are present in pro- and eukaryotes, enzymes of the DMSO reductase family are exclusively present in prokaryotes. The molybdenum cofactor (Moco) in these group of enzymes is present in the form of the bis-molybdopterin guanine dinucleotide (bis-MGD) cofactor [3], generally coordinating two molybdopterin guanine dinucleotide moieties in addition to a sulfido-/oxo-/ or selenido ligand and an amino acid ligand from the protein which can be a serine, cysteine, selenocysteine or aspartate. Further, the enzymes can contain additional cofactors such as iron-sulfur (Fe-S) clusters, cytochromes or flavin nucleotide cofactors [4]. Well-characterized examples of the DMSO reductase family include subfamily enzymes such as DMSO reductases, trimethylamine *N*-oxide (TMAO) reductases, formate dehydrogenases (FDH) or nitrate reductases (NR) [1]. The crystal structures of these enzymes revealed that the bis-MGD cofactor is deeply buried inside the proteins, at the end of a funnel-shaped passage giving access only to the substrate [5]. The localization of the cofactor within the enzyme suggested that chaperones are required to facilitate the insertion of the complex bis-MGD cofactor into their active sites by the involvement of a final folding step of the target enzyme after bis-MGD insertion [6–13]. For enzymes of the DMSO reductase family, these chaperones are also referred to as redox enzyme maturation proteins (REMPs) [14]. In general, these chaperones are highly specific for their target enzyme [15]. The chaperones for the DMSO reductase family enzymes were divided into different subfamilies: the NarJ-like chaperones for nitrate reductases, the DmsD-like chaperones for periplasmic DMSO reductases, the TorD-like chaperones for TMAO reductases, the YcdY-like chaperones for maturation of the YcdX proteins, and the FdhD-like chaperones for the maturation of several distinct FDHs [13, 16–18].

For the FdhD-subfamily, well-characterized representatives comprise *Escherichia coli* FdhD and *Rhodobacter capsulatus* FdsC [19–21]. FdhD displays an exception of the general proposed rule that all molybdochaperones are specific for one target molybdoenzyme [14], since the FdhD is essential for the maturation of all three *E*. *coli* FDHs, namely FdhF, FdoGHI, and FdnGHI [20]. The three *E*. *coli* FDH enzymes have in common that their catalytic subunits coordinate the bis-MGD cofactor with a selenocysteine ligand and a sulfido-ligand, and as additional cofactor a [4Fe-4S] cluster that is present in vicinity of the bis-MGD moiety [22, 23]. Additional subunits and the localization of the enzymes within the cell vary. FdnGHI and FdoGHI show high sequence similarities, both enzymes are trimers facing the periplasm that are membrane-bound via the I-subunit [24, 25]. The electrons are transferred from the substrate converted in the catalytic subunit (FdnG or FdoG) via the five Fe-S cluster-containing subunit (FdnH or FdoH) to the membrane anchoring cytochrome-containing subunit (FdnI or FdoI) finally to the quinone-pool [26]. FdnGHI expression is induced in the presence of nitrate under anaerobic conditions. In contrast, FdoGHI is also present at low levels under aerobiosis, during fermentative conditions and during nitrate respiration [26, 27]. The third FDH in *E*. *coli*, FdhF is facing the cytoplasm and forms the formate hydrogen lyase (FHL) complex together with the hydrogenase-3 (encoded by *hycBCDEFG*) [28]. The FHL complex is membrane bound and produces H_2_ and CO_2_ under fermentative conditions [29]. The expression of the gene is repressed by the presence of nitrate [29]. Recently, the role of the specific chaperone FdhD was investigated in detail for FdhF maturation, and it was shown that FdhD is essential for the insertion of the terminal sulfido-ligand present at the bis-MGD cofactor of FdhF in addition to FdnG and FdoG [19, 20]. FdhF was inactive when FdhD was absent during expression, but after purification the enzyme could be activated by chemical sulfuration [20]. For sulfuration of bis-MGD, FdhD specifically interacts with the l-cysteine desulfurase IscS in *E*. *coli* [20]. It has been suggested that IscS transfers the sulfur from l-cysteine to FdhD in form of a persulfide. The cysteines 121 and 124 located in a conserved CXXC motif of FdhD were proposed in the studies by Thomé et al. [20] and Arnoux et al. [19] to be involved in the sulfur transfer process from IscS to bis-MGD. Cysteine to alanine variants in these residues were shown to produce an inactive FdhF enzyme. FdhD was co-crystallized in complex with GDP and direct binding of bis-MGD to FdhD was suggested [19], but it has not been proven so far that the cofactor is bound in an active form.

Previously, the binding of bis-MGD to the FdhD-like chaperone FdsC from *R*. *capsulatus* has been investigated in detail [21]. Using *R*. *capsulatus* FdsC as source of bis-MGD cofactor in a direct reconstitution assay, it was shown that an intact bis-MGD cofactor was inserted into *E*. *coli* TMAO reductase (TorA) that reconstituted TorA activity [21]. In contrast to *E*. *coli*, *R*. *capsulatus* harbors only one FDH enzyme that is encoded by the *fdsGBACD* operon [30]. The enzyme was shown to be structurally different from the *E*. *coli* FDH enzymes, since a soluble (FdsGBA)_2_ heterodimer is formed that is located in the cytoplasm. The purified enzyme was shown to be oxygen tolerant using NAD^+^ as terminal electron acceptor. However, the physiological role of this enzyme has not been characterized to date. Previous studies showed that the (FdsGBA)_2_ enzyme expressed in the absence of FdsC contained a bis-MGD cofactor that lacked the terminal sulfido group [31, 32]. However, in contrast to *E*. *coli* FdhD, the cysteines 104 and 107 in FdsC (corresponding to Cys121 and Cys107 in FdhD) were not essential for the production of an active *R*. *capsulatus* FDH [21]. In *R*. *capsulatus*, three l-cysteine desulfurases are present: NifS2, NifS3 and NifS4 [33]. While the specific role of all three enzymes has not been characterized in detail so far, NifS4 was shown to be involved in the mobilization and transfer of sulfur to the Moco present in *R*. *capsulatus* xanthine dehydrogenase [33].

In this study, we compared the roles of FdhD and FdsC in the maturation of FDH enzymes and investigated the roles of the conserved cysteines in the conserved CXXC motif in this process. Since FdhD and FdsC share an amino acid sequence similarity of 46.3% and an identity of 32.4%, both chaperones were suggested to be functional homologues. We show that FdhD and FdsC have a common role that is not only specific to the FDH enzymes present in the respective organism. FdsC and FdhD can be interchanged on structurally very distinct FDH enzymes. Further, we demonstrate that the cysteine residues in the motif CXXC are not essential for the production of active FDH. Solely, FdhD-C121 was revealed to have a particular role in stimulating the activity of *E*. *coli* IscS exclusively. No similar effect was identified for *R*. *capsulatus* FdsC. We suggest that FdhD-Cys121 is assisting the specific interaction with *E*. *coli* IscS and enhances its l-cysteine desulfurase activity. By this process a higher sulfuration and transfer efficiency of bis-MGD is obtained, which might be only required for the *E*. *coli* system, since *E*. *coli* IscS has numerous interaction partners.

## Results

### A phylogenetic view of the FdhD family of molecular chaperones for FDH enzymes

**Fig 1:**
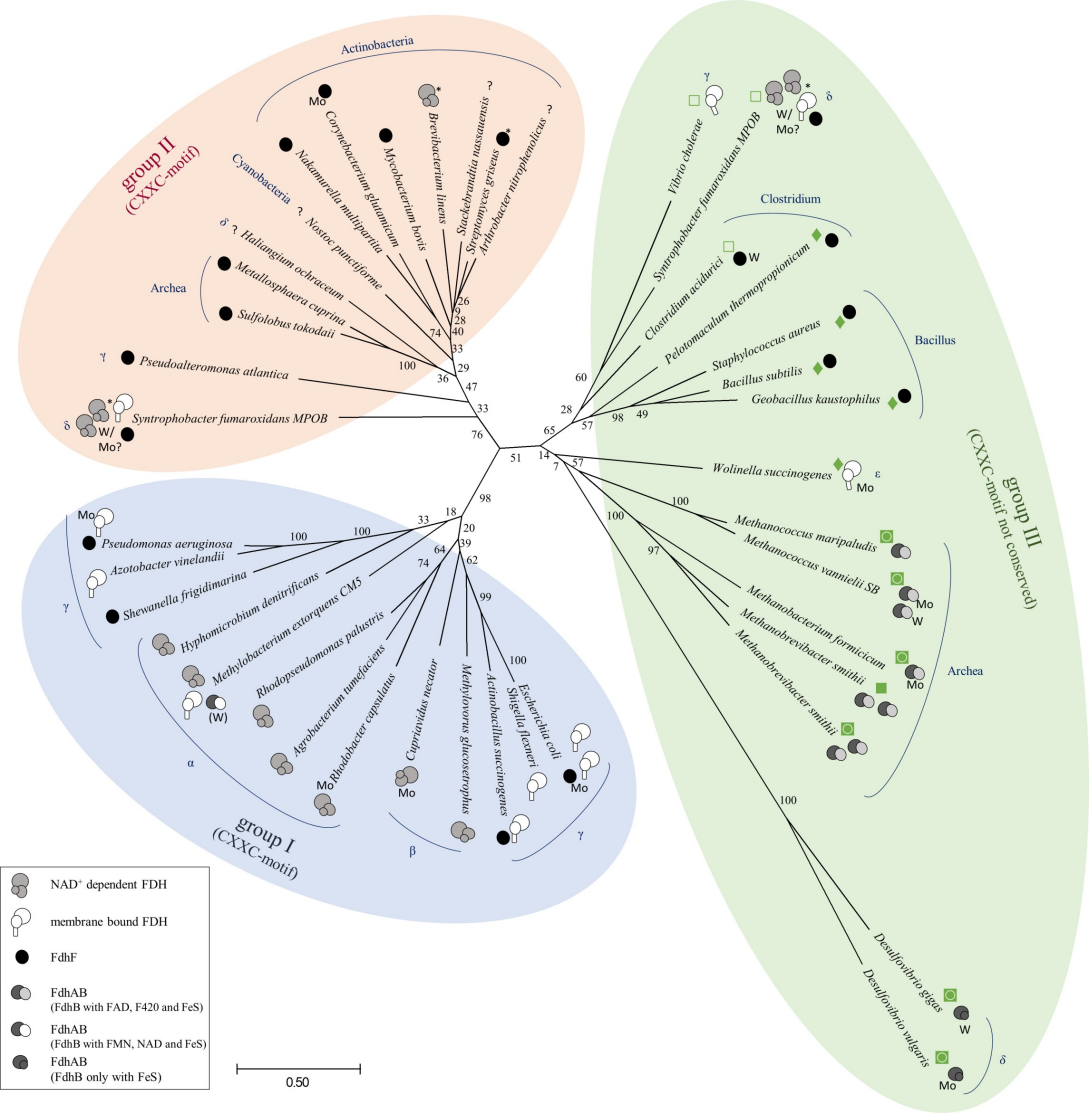
Phylogenetic tree of FdhD-like proteins. Protein phylogeny of FdsC/FdhD homologues based on a full length multiple sequence alignment by Muscle [50]. The tree was constructed using the Maximum Likelihood method based on the Dayhoff matrix based model [51][57]. The tree is drawn to scale, with branch lengths in the same units as those of the evolutionary distances used to infer the phylogenetic tree. The evolutionary distances were computed using the Dayhoff matrix based method and are in the units of the number of amino acid substitutions per site [52]. The scale bar indicates 0.2 substitutions per site. Numbers near branches indicate the bootstrap proportion for 100 replicas using the same method. The analysis involved 41 amino acid sequences. All positions containing gaps and missing data were eliminated. There was a total of 174 positions in the final dataset. Evolutionary analyses were conducted in MEGA7 [53]. Domains and classes of prokaryotes are marked in blue: α- Proteobacteria, β-Proteobacteria, γ-Proteobacteria, δ-Proteobacteria, ε-Proteobacteria. There are three main branches: group I, II and III. FdsC/FdhD homologues in group I and II harbor the conserved CXXC-motif, which is not present in group III (♦ - CC-motif, □ - only second cysteine, ■ - CXXXCXC-motif, ◙ - no cysteine). Existing FDHs are listed as described in the box on the left and based on genomic analysis using www.ncbi.nlm.nih.gov or * img.jgi.doe.gov. If already described in literature, the corresponding active site metal (Mo or W) is displayed.

Analysis of bacterial and archaeal genome sequence data in 5 phyla and 33 taxonomic families revealed a wide distribution of homologues to the *E*. *coli* FdhD protein (referred to as FdhD family of chaperones) (Fig 1). In bacteria like *E*. *coli*, where three FDH enzymes are present, only one FdhD-homologue has been identified. We aligned 41 FdhD-like sequences, which present an overall level of identity of about 18–34%. From the amino acid sequence alignment, an unrooted phylogenetic tree has been constructed (Fig 1). Overall, the phylogenetic tree can be divided into three groups. In group I only α-, β- and γ-proteobacterial FdhDs are present, including the ones from *E*. *coli* and *R*. *capsulatus*. Group II contains mainly FdhDs from Actinobacteria, but also from Cyanobacteria, Archaea and γ- or δ-Proteobacteria. Group III contains a larger group of archaeal FdhDs in addition to FdhD proteins from Bacilli and Clostridia or γ-, δ-, and ε- Proteobacteria. Overall, Firmicutes, Proteobacteria and Actinobacteria are known to contain a high to medium number of respiratory molybdoenzymes [34] which is obvious by the distribution of FdhD homologues in these organisms. Interestingly, γ-Proteobacteria are represented in all three groups. Many pathogens belong to this class of bacteria which are known to harbor a high number of molybdoenzymes representing a higher metabolic flexibility [34]. Thus, a wide distribution of FdhD-like chaperones is found here. To provide more details, we also tried to include the different target FDH enzymes in the phylogenetic tree, to analyze whether FdhD has evolved together with its protein partner [14]. Here, we divided the FDH enzymes into membrane-bound FdhF-type, dimeric FdhAB-type, and cytosolic NAD^+^-dependent FDHs. Unfortunately, a lot of FDHs are not characterized to date so that our division is mainly based on amino acid sequence homologies of characterized FDH enzymes. The overall distribution revealed that the FdhD homologues for NAD^+^-dependent FDHs are mainly present in group I, while group II predominantly contains FdhDs required for monomeric FdhF-type enzymes. Group III can be divided into two subgroups, while one subgroup contains mainly FdhD-homologues for FdhF-type enzymes, the second subgroup predominantly contains FdhD proteins for FdhAB-type FDHs. A previous study reporting on the evolution of the chaperones for the DMSO reductase family enzymes suggested that the molecular chaperones evolved together with its molybdenum partner protein [14]. However, a division into FdhDs for Mo-or W-containing FDHs was not possible, and FdhD proteins acting on either Mo- or W-containing (or both) FDHs are distributed in all three groups of the phylogenetic tree without any obvious division.

It has been reported previously that FdhD proteins are characterized by a highly conserved CXXC motif that is present on a disordered loop of the protein [19]. In *E*. *coli* FdhD, the cysteines 121 and 124 of this motif have been reported to be functionally important for the catalytic sulfur transferring activity from IscS for the formation of a sulfurated bis-MGD cofactor [20]. However, as obvious from the data in the phylogenetic tree, a detailed analysis of the amino acid sequence of the FdhD-like proteins showed that the CXXC motif is not highly conserved. FdhD proteins containing the CXXC motif are mainly present in group I and group II of the phylogenetic tree. Group III, in contrast, contains mainly FdhD sequences in which either only the second cysteine of the motif is conserved, which have a CC-motif instead or have no cysteines. Thus, this puzzling point encouraged us to understand the importance of the cysteines for the role of FdhD-like proteins from *E*. *coli* and *R*. *capsulatus*.

### Copurification of *E*. *coli* FdhD and *R*. *capsulatus* FdsC variants with bis-MGD

**Fig 2:**
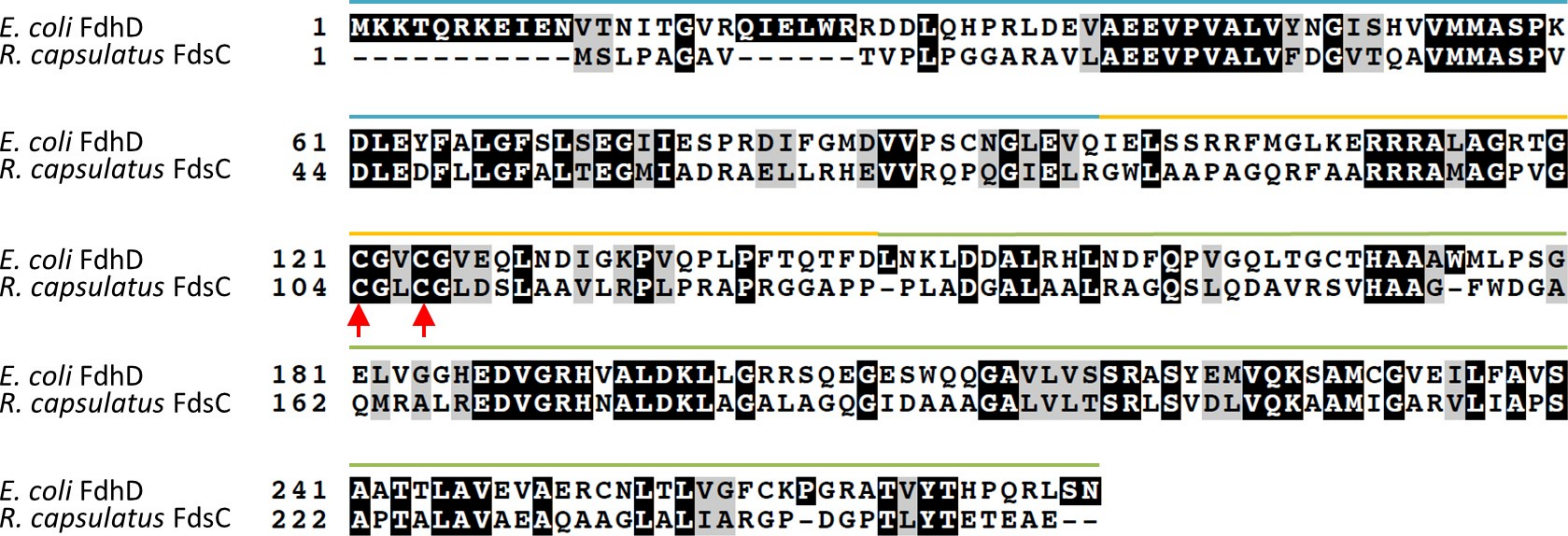
Amino acid sequence alignment of *E*. *coli* FdhD and *R*. *capsulatus* FdsC. The amino acid sequence alignment was generated using ClustalW. Identical amino acids and similar amino acids are marked with a black and a grey box, respectively. The conserved cysteine residues are labelled with red arrows. The blue bar over the alignment indicates the N-terminal domain (blue), the linker region (yellow) and the C-terminal domain (green) based on the structure of FdhD (PDB: 4pde) [19].

For *E*. *coli* FdhD, it has been reported that especially the first cysteine of the two cysteines in the conserved ^121^CXXC^124^ motif is essential for its role in transferring the sulfur from IscS to the bis-MGD cofactor, while Cys124 is additionally required for the production of an active FdhF enzyme [20]. In contrast, it has been shown for *R*. *capsulatus* FdsC that the two conserved cysteine residues are not essential for producing an active sulfido-containing (FdsGBA)_2_ enzyme [21]. To test whether the role of the cysteines is specific for the *E*. *coli* FDH enzymes, which are structurally and functionally different from the soluble *R*. *capsulatus* (FdsGBA)_2_ enzyme, we constructed single and double cysteine variants of *E*. *coli* FdhD (C121A, C124A, C121A/C124A) and compared their characteristics to *R*. *capsulatus* FdsC. For *R*. *capsulatus* FdsC the corresponding cysteine variants were already available (C104A, C107A, C104A/C107A) [21]. To directly compare the role of the two chaperones, the three cysteine variants of both FdsC and FdhD in addition to the wild-type protein were purified from an *E*. *coli ΔfdhD* strain. An amino acid sequence alignment of both proteins is shown in Fig 2, highlighting the position of the cysteines within the protein sequences.

**Fig 3:**
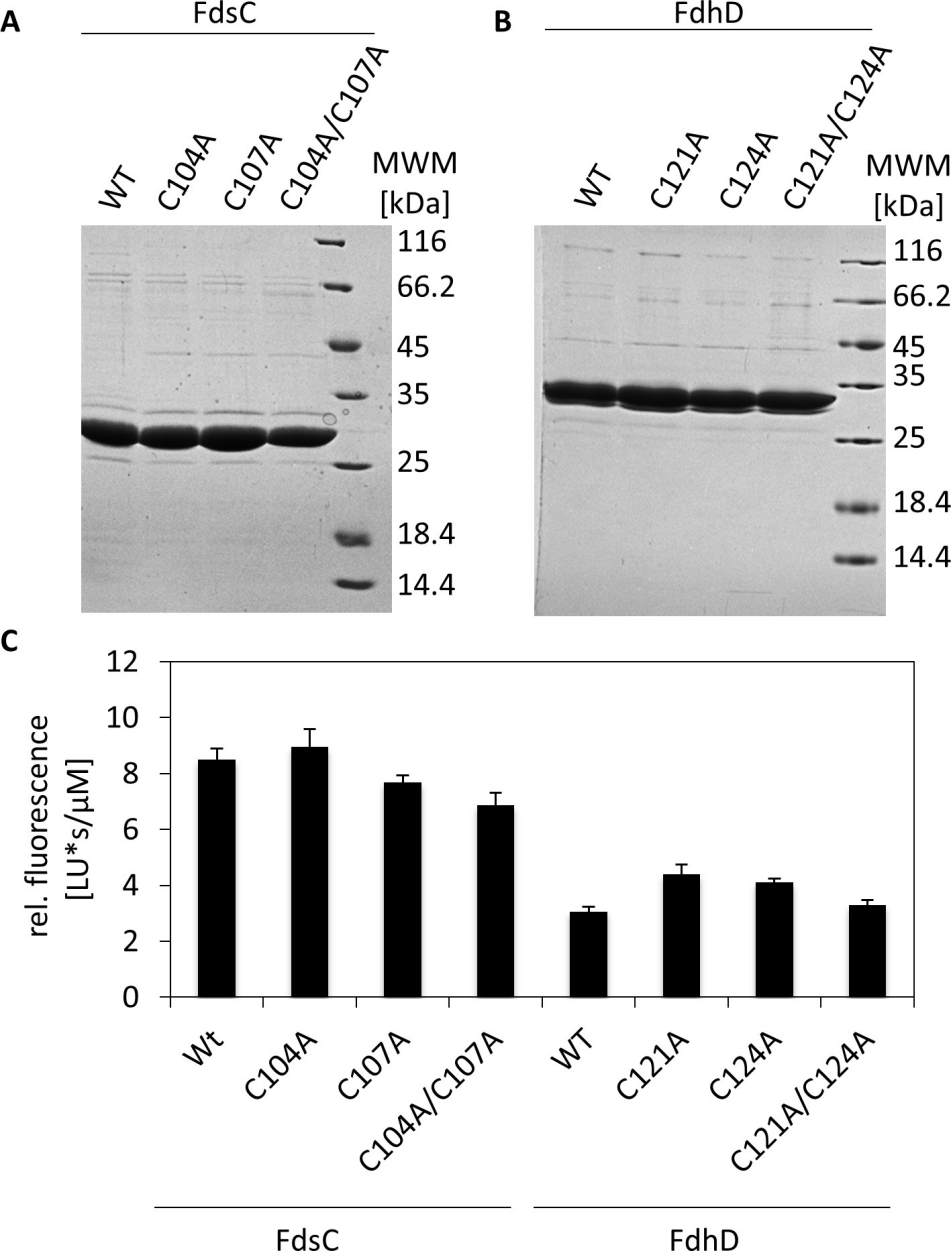
Characterization of cysteine variants of FdsC and FdhD. FdsC and FdhD and their cysteine variants were expressed in *E*. *coli ΔfdhD* strain and purified by Ni-NTA chromatography as described for FdsC previously [21]. A) 15 μg of purified FdsC, FdsC-C104A, FdsC-C107A and FdsC-C104/C107A were separated by SDS-PAGE. B) 15 μg of purified FdhD, FdhD-C121A, FdhD-C124A and FdhD-C121/C124A were separated by SDS-PAGE. C) 200–300 μM of protein was treated with I_2_/KI-HCl to isolate Form A-GMP as described previously [47]. Form A-GMP was separated by HPLC. The corresponding peak area was normalized for protein concentration. The results represent the mean values from three independent measurements (±S.D.).

The proteins were purified by Ni-NTA chromatography and the Coomassie stained SDS-gels showed that all eight proteins were purified with a purity of more than 90%. His_6_-tagged FdhD has a molecular mass of 32.7 kDa, while His_6_-tagged FdsC has a molecular mass of 28.6 kDa (Fig 3A and 3B). Size exclusion chromatography confirmed that all FdsC and FdhD variants were purified as a dimer in solution (data not shown). For FdsC it has been shown before that the protein was copurified with bound bis-MGD cofactor under our expression conditions [21]. To analyze the bis-MGD content for all FdsC and FdhD variants, the bis-MGD cofactor was extracted from the respective protein, oxidized by I_2_/KI overnight at room temperature, and the obtained fluorescent derivative FormA-GMP was quantified after separation on a reversed-phase C18 HPLC column. The results in Fig 3C show that all FdsC and FdhD variants were copurified with the bis-MGD cofactor, however, the bis-MGD content of the FdsC wild type and variants was in average twice as high in comparison to the FdhD proteins. The bis-MGD binding ability thereby was not influenced by the cysteine to alanine exchanges in the variants.

### Reconstitution of TMAO reductase activity with bis-MGD provided by FdsC or FdhD

An *in vitro* assay system has been established previously by which the direct insertion of the bis-MGD cofactor bound to FdsC into apo-TorA can be monitored [21]. Very recent results showed that TorA is only active when the bis-MGD cofactor contains a terminal sulfido group [35]. Thus, the *in vitro* reconstitution of TorA activity can be used to determine the sulfuration level of the bis-MGD cofactor bound to FdhD or FdsC.

**Fig 4:**
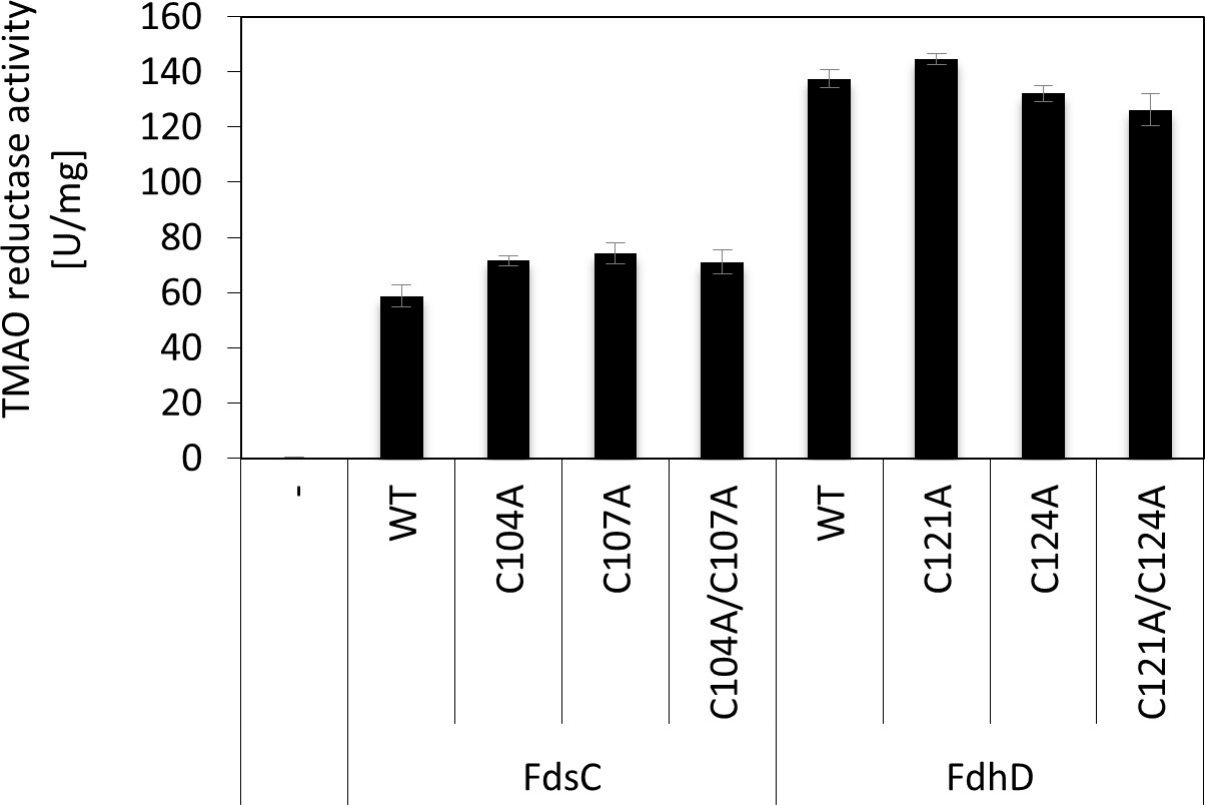
Reconstitution of *E*. *coli* apo-TMAO reductase using FdsC or FdhD as bis-MGD source. 50 μM of each purified chaperone variant was incubated with apo-TMAO reductase (8 μM) to reconstitute enzyme activity. After 2 hours TMAO reductase activity was measured under anaerobic conditions following the oxidation of reduced benzyl viologen at 600 nm in the presence of 0.1 μM TMAO. Kinetic Data are mean values from three independent measurements (±S.D.).

For the *in vitro* reconstitution assay, 8 **μ**M of Moco-deficient apo-TorA were incubated with 50 **μ**M of the FdhD or FdsC-variants and incubated for 2 hours at 37°C under anaerobic conditions without any further supplementations. The results in Fig 4 show that active TorA was obtained for both the FdhD and FdsC variants. Unexpectedly, while the TorA activity varied around 60–80 U/mg when FdsC wild-type or the C104A, C107A or C104A/C107A variants were used as bis-MGD source, almost 2-fold higher TorA activities were obtained with FdhD and the variants C121A, C124A or C121A/C124A with values around 130–150 U/mg. No clear differences in activity were obtained between FdsC and the cysteine substitution variants or FdhD and the cysteine substitution variants.

**Fig 5:**
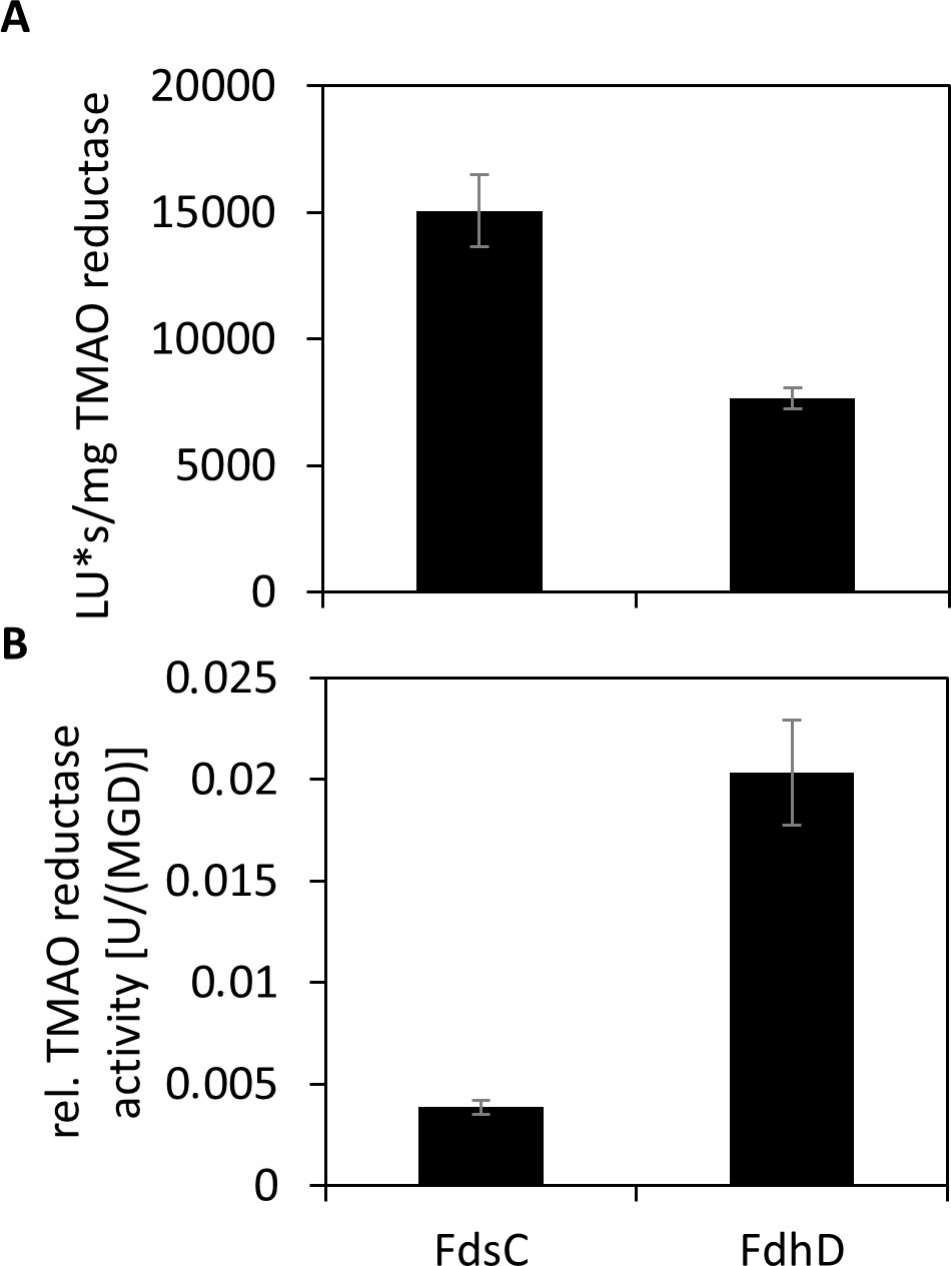
Characterization of *E*. *coli* apo-TMAO reductase reconstituted with FdsC or FdhD. For the reconstitution of enzyme activity, 50 μM of each FdhD, FdsC or their variants was incubated with apo-TMAO reductase (8 μM) in a total volume of 8 ml. After 7 hours, the samples were concentrated to 500 μl before size exclusion chromatography. The results represent the mean values from three independent measurements (±S.D.). A) Relative Form A-GMP content (MGD in LU*s per mg TMAO reductase) of 3 μM TMAO reductase was analyzed by HPLC. B) Activity of the fraction containing TMAO reductase was measured under anaerobic conditions following the oxidation of reduced benzyl viologen at 600 nm in the presence of 0.1 μM TMAO. TMAO reductase activity was normalized for the MGD content.

Since the results in Fig 3C showed that overall, FdhD and the C121A, C124A or C121A/C124A variants were loaded with lower levels of bis-MGD, we compared the bis-MGD content of the reconstituted TorA proteins using FdhD or FdsC proteins. We analyzed only the bis-MGD loading of TorA after reconstitution using FdsC and FdhD wild-type proteins, since the analysis requires a lot of source TorA protein for bis-MGD quantification and differences were not expected between the wild-type and the cysteine variants according to Fig 3. FdsC-reconstituted TorA contained about two-times the amount of bis-MGD in comparison to FdhD-reconstituted TorA (Fig 5A). However, when the obtained specific TMAO reductase activities from Fig 4 of FdsC and FdhD wild-type were related to their bis-MGD levels (detected as FormA-GMP), it became obvious that about 5-fold higher relative TorA activity in relation to bis-MGD bound to the protein was obtained with FdhD in comparison to FdsC. Since both proteins bind the bis-MGD cofactor, the differences in activity can only be explained by a different saturation level of the sulfido-ligand at the molybdenum atom. In a recent report by Kaufmann et al. [35], it was shown that the presence of a terminal sulfido-ligand at the active site of TorA contributed to a 20-fold increased TorA activity. In contrast, enzyme preparations containing an oxo-ligand instead were mainly inactive. Therefore, it has to be concluded that the bis-MGD cofactor on FdhD is more saturated with the sulfido-ligand than the cofactor present on FdsC, which of course can only be qualitatively speculated from this assay.

### Influence of FdhD and FdsC variants on the maturation of FDH enzymes from different classes

So far, the results showed that the cysteine substitutions in either FdsC or FdhD did not influence the bis-MGD binding ability or transfer of the cofactor to TorA. For *E*. *coli* FdhD, however, it has been reported before that Cys121 and Cys124 are essential for the production of an active FdhF enzyme [20]. In contrast, for the corresponding C104A and C107A substitution variants of *R*. *capsulatus* FdsC, no effect on (FdsGBA)_2_ activity was reported, using a heterologous expression system in *E*. *coli* [21]. To differentiate whether the observed differences in the role of the cysteine residue is based on differences in the chaperone or the type of FDH enzyme used, we analyzed the effect of *E*. *coli* FdhD on *R*. *capsulatus* (FdsGBA)_2_ activity and of *R*. *capsulatus* FdsC on *E*. *coli* (FdoGHI)_3_ activity.

**Fig 6:**
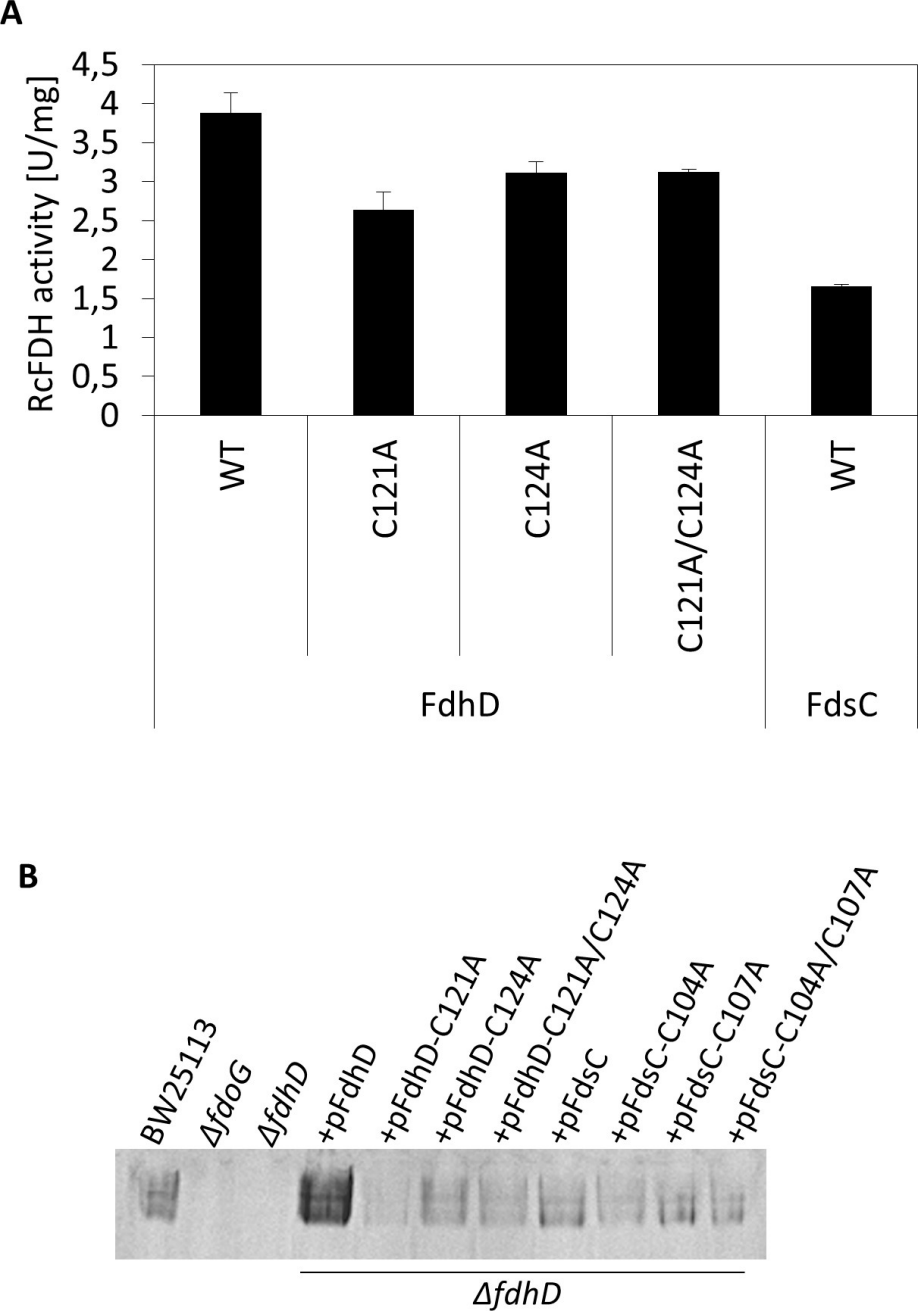
Influence of FdsC and FdhD on FDH activity. A) *R*. *capsulatus* (FdsGBA)_2_ was expressed in *ΔfdhD* strain in the presence of FdhD WT (pNB14), FdhD-C121A (pNB15), FdhD-C124A (pNB16), FdhD-C121A/C124A (pNB17) or FdsC WT (pTHfds03) and purified by Ni-NTA affinity chromatography as described previously [21]. *R*. *capsulatus* (FdsGBA)_2_ activity was detected photometrically by the increase in NADH recorded at 340 nm and 1 U/mg is defined as the reduction of 1 μmol NAD^+^/min/mg of enzyme at room temperature. Kinetic Data are mean values from three independent measurements (±S.D.).The enzyme expressed in the absence of FdsC or FdhD was not active (data not shown), as reported previously [21]. B) 55 ml cultures of each strain were grown anaerobically at 37°C for 16 hours in LB media in the presence of 20 μM IPTG, 10 μM sodium molybdate and antibiotic as needed. Equivalent amounts (40–60 μg) of Triton-X100 treated crude extracts were applied to each lane and separated by non-denaturing PAGE. The gels were stained with 1 mM NBT, 0.5 mM PMS and 50 mM formate in 50 mM potassium phosphate, pH 6.8. Lanes from left to right: BW25113, *ΔfdoG*, *ΔfdhD*, +pFdhD: *ΔfdhD* deficient strain complemented with plasmid pNB14, +pFdhD-C121A: *ΔfdhD* deficient strain complemented with plasmid pNB15, +pFdhD-C124A: *ΔfdhD* deficient strain complemented with plasmid pNB16, +pFdhD-C121A/C124A: *ΔfdhD* deficient strain complemented with plasmid pNB15, +pFdsC: *ΔfdhD* deficient strain complemented with plasmid pTHfds14, +pFdsC-C104A: *ΔfdhD* deficient strain complemented with plasmid pNBfds04, +pFdsC-C107A: *ΔfdhD* deficient strain complemented with plasmid pNBfds05, +pFdsC-C104A/C107A: *ΔfdhD* deficient strain complemented with plasmid pNBfds06.

Fig 6 shows the results on *R*. *capsulatus* (FdsGBA)_2_ activity after coexpression with *E*. *coli* FdhD wild-type or the cysteine variants in an *E*. *coli ΔfdhD* deletion strain. The results show that active *R*. *capsulatus* (FdsGBA)_2_ enzymes were obtained, showing that *E*. *coli* FdhD can replace FdsC in its function. Surprisingly, the FDH activity was even 2.6-fold higher with FdhD wild-type in comparison to FdsC wild-type. When the cysteine variants C121A, C124A or the double variant C121A/C124A of FdhD were coexpressed instead, a 1.4 times lower activity was obtained in comparison to FdhD wild-type, showing that the cysteine substitutions indeed have an effect on the role of FdhD for the maturation of (FdsGBA)_2_. However, the results show that FdhD-C121 or FdhD-C124 are not essential for the maturation of the *R*. *capsulatus* enzyme, since FDH activity was obtained. Rather, the presence of both cysteines was required to obtain the highest FDH activity.

For comparison, the roles of the cysteines in FdhD were additionally analyzed for *E*. *coli* (FdoGHI)_3_ activity. Here, we made use of an in-gel activity assay that directly detects the activity of (FdoGHI)_3_ after separation in native gels. To suppress the activity of (FdnGHI)_3_, cells were grown with 0.2% sodium formate in the absence of nitrate. The gel was incubated at 37°C under aerobic conditions with a staining solution containing sodium formate, NBT and PMS.

The (FdoGHI)_3_ activity obtained after expression of FdhD wild-type or the C121A, C124A, C121A/C124A variants in an *E*. *coli ΔfdhD* strain showed that the highest activity was obtained with the FdhD wild-type protein (Fig 6A). In contradiction to previous reports, we obtained an (FdoGHI)_3_ activity when the FdhD variants C121A, C124A or the double variant C121A/C124A were used [19, 20]. While the in-gel activities of the FdhD-C124A and the FdhD-C121A/C124A variants were comparable, the FdhD-C121A variant showed a slightly reduced activity (Fig 5A). However, since the FdhD-C121A/C1214A double variant was active, the results show that Cys121 is not essential for FdoGHI activity.

In contrast to FdhD, the cysteine variants of *R*. *capsulatus* FdsC, C104A, C107A, and the double variant C104A/C107A showed no difference on FdoGHI activity in comparison to FdsC wild-type (Fig 6B). Additionally, the in-gel activities were overall comparable to the FdhD cysteine variants. These results reveal that differences in the maturation of FdoGHI exist between FdsC and FdhD wild-type proteins. However, when the cysteines in FdhD were substituted, the maturation efficiency of the FdhD variants was comparable to FdsC. This implies that the cysteines in FdhD have a role that is specific to the FdhD protein.

### Influence of FdhD and FdsC on the l-cysteine desulfurase activity of *E*. *coli* IscS

**Fig 7:**
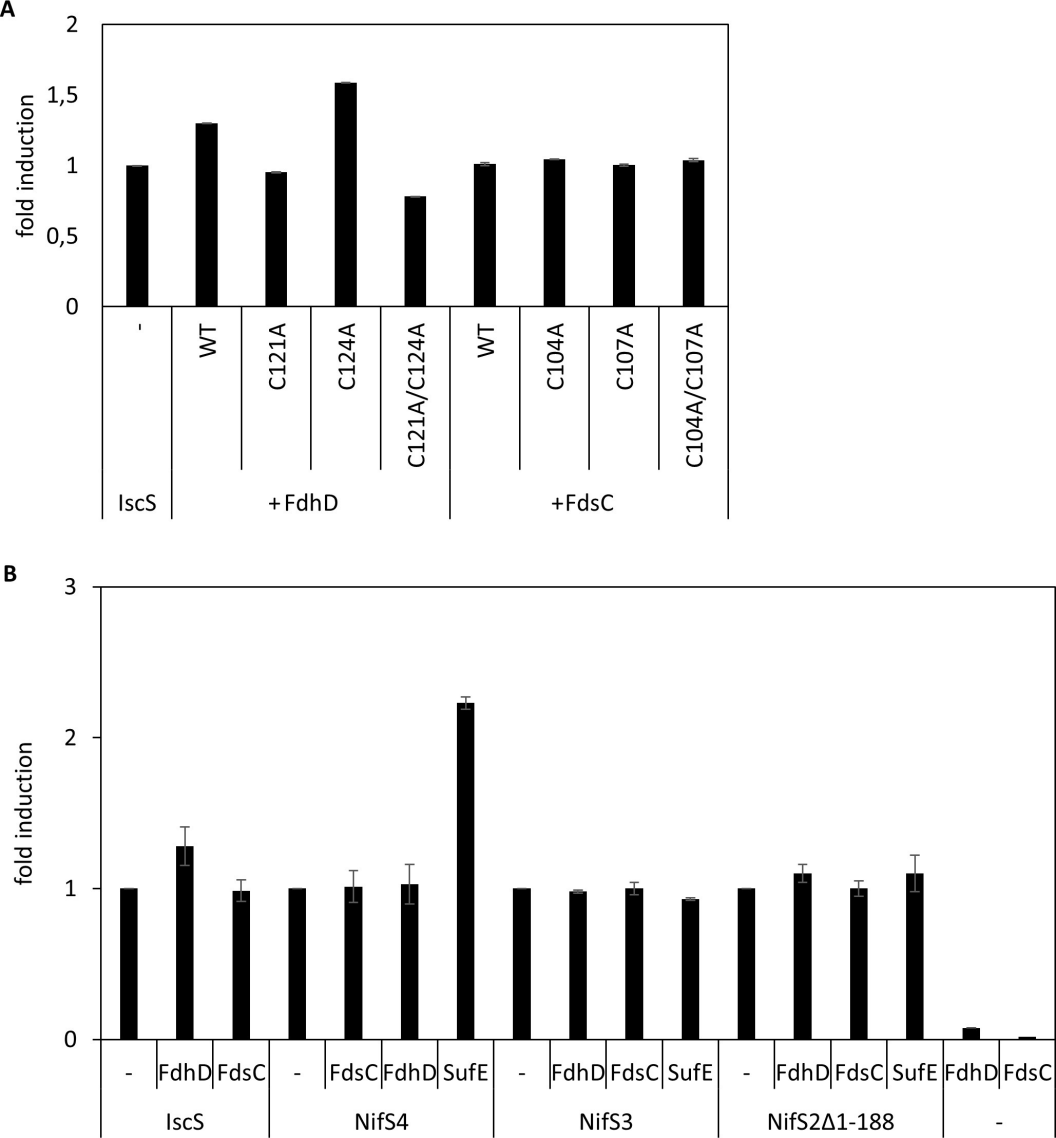
Influence of FdsC and FdhD on l-cysteine desulfurase activity. l-cysteine desulfurase activity was measured by determination of total sulfide produced [48]. A) 1 μM IscS from *E*. *coli* was mixed with either 2 μM FdhD/FdhD variants or FdsC/FdsC variants and incubated for 10 min in the presence of 1 mM l-cysteine at 30°C. The activity of IscS alone was set to 1. B) l-cysteine desulfurase IscS (1 μM) from *E*. *coli* or NifS4, NifS3 and NifS2-Δ1–188 from *R*. *capsulatus* (2 μM) were mixed in a 1:2 ratio with FdsC, FdhD or SufE, respectively and incubated for 10 min in the presence of 1 mM l-cysteine at 30°C. The fold induction of IscS, NifS4, NifS3 or NifS2-Δ1–188 activity by incubation with FdsC, FdhD or SufE is relative to the activity of the l-cysteine desulfurase alone, respectively. Kinetic Data are mean values from three independent measurements (±S.D.).

The results shown above indicated that the enhanced (FdoGHI)_3_ activity by FdhD wild-type in comparison to its cysteine variants might be based on an enhancing effect of FdhD on the l-cysteine desulfurase activity of IscS. IscS was shown before to provide the sulfur for the formation of the sulfido ligand on the bis-MGD cofactor. It has been reported previously that FdhD stimulates the activity of IscS, while this stimulating effect was lost in the FdhD-C121A single and FdhD-C121A/C124A double variants [19, 20]. We were able to reproduce these results (Fig 7A), however, when we tested the effect of *R*. *capsulatus* FdsC on IscS activity, we did not observe a stimulating effect (Fig 7A). Further, we tested the effect of both FdhD and FdsC on the l-cysteine desulfurase proteins present in *R*. *capsulatus*. *R*. *capsulatus* contains the three l-cysteine desulfurases NifS2, NifS3 and NifS4 as house-keeping l-cysteine desulfurases, while a fourth one, NifS, was shown to be specific for nitrogenase [33]. The results in Fig 7B show that neither FdhD nor FdsC could stimulate the l-cysteine desulfurase activity of any of the three *R*. *capsulatus* l-cysteine desulfurases NifS2, NifS3 or NifS4. A truncated version of Nfs2 was used, since the full-length protein was shown to be unstable [33]. As positive control, we used the *R*. *capsulatus* SufE protein, the physiological partner of NifS4 that was able to stimulate the activity of NifS4 but not of the other l-cysteine desulfurases. The results imply that FdhD-C121 is specifically required for stimulating the activity of *E*. *coli* IscS.

### Interaction of FdhD and FdsC with l-cysteine desulfurases

It has been reported previously that FdhD forms a complex with IscS that can be copurified [19, 20]. To analyze whether the FdhD-C121A/C124A variant effects the interaction with IscS, we performed analytical size exclusion chromatography. For comparison, we also analyzed the complex formation of IscS with FdsC and the FdsC-C104A/C107A variant.

**Fig 8:**
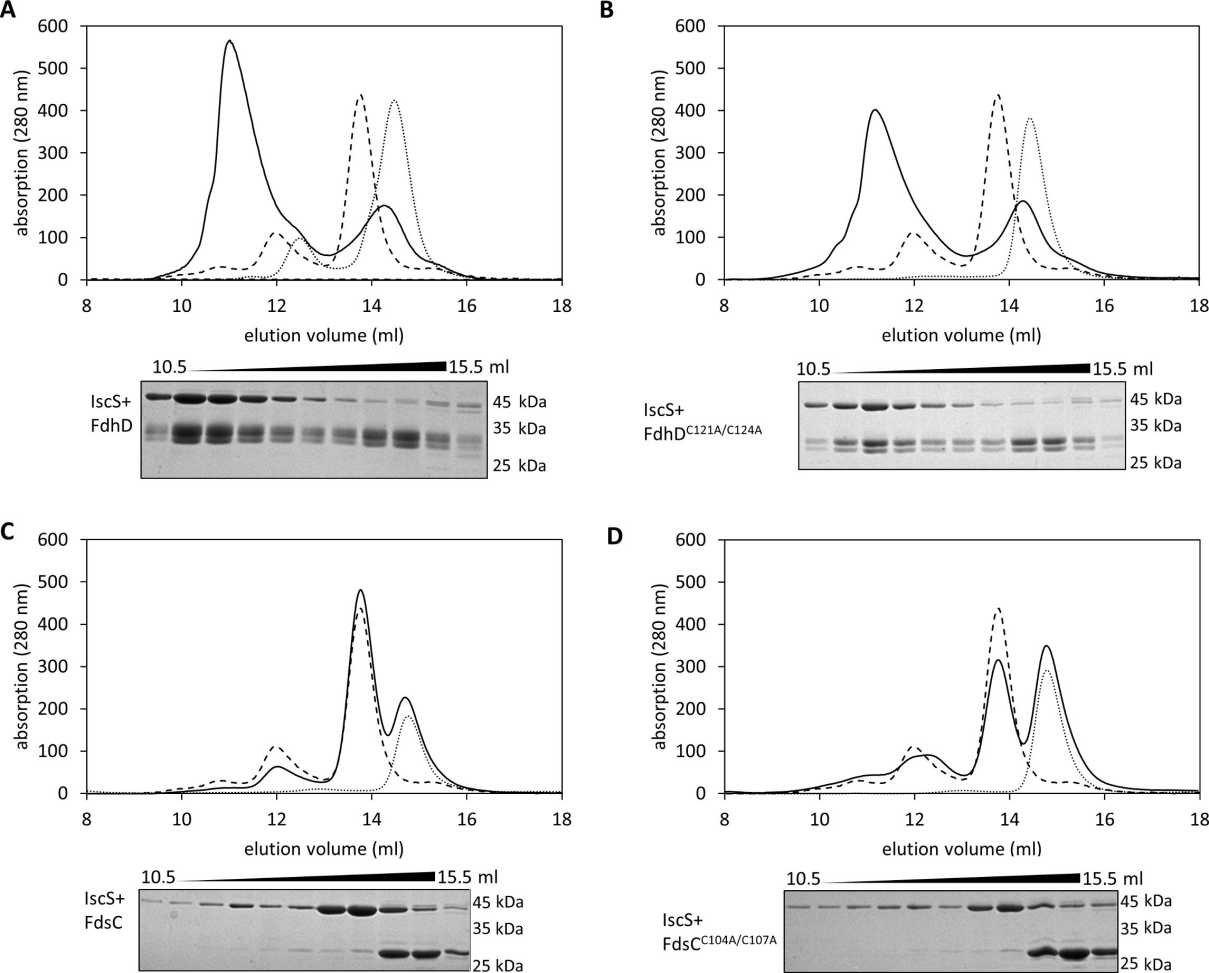
Interaction of FdhD with IscS. Complex formation of (A) 40 μM FdhD + 20 μM IscS, (B) 40 μM FdhD-C121A/C124A + 20 μM IscS, (C) 40 μM FdsC + 20 μM IscS and (D) 40 μM FdsC-C104A/C107A + 20 μM IscS were analyzed by size exclusion chromatography on a Superdex Increase 200 column (GE Healthcare) equilibrated in 100 mM potassium phosphate buffer, pH 8.0, 100 mM NaCl, 10 mM β-mercaptoethanol. The elution of proteins was followed at 280 nm. Indicated fractions in a range of 10.5–15.5 ml (0.5 ml fractions) were analyzed for their protein content by 15%SDS−PAGE.

The chaperones FdhD or FdsC were incubated with IscS and complex formation was analyzed after separation on a Superdex 200 column. When a complex between IscS and one of the chaperones is formed, the complex elutes more rapidly from the size exclusion column due to its increase in molecular mass. A 1:1 complex between the IscS dimer and FdhD is expected, as reported previously [19, 20]. The results in Fig 8A show that FdhD and IscS readily formed a complex that co-eluted at 11 ml after separation on a Superdex 200 column. The peak fractions eluting 10–12 ml were separated by SDS-PAGE and showed the presence of both proteins, confirming the formation of a protein-complex. The complex formation was not impaired when using the FdhD-C121A/C124A variant showing that the two cysteines are not essential for complex formation, since a comparable elution volume of 11 ml was obtained (Fig 8B). In contrast, no complex formation was determined between IscS and FdsC or the FdsC-C104A/C107A variant (Fig 8C and 8D), as obvious from the elution profile and the corresponding fractions separated by SDS-PAGE, since no peak with a different elution time was obtained after coincubation of the proteins. The results therefore reveal that FdhD forms complex with IscS that can be copurified, in contrast to FdsC, where no stable complex with IscS was formed.

## Discussion

FdhD-like chaperones are found in a vast number of prokaryotes. They are often encoded in the same operon together with the structural genes for FDH enzymes or in vicinity to genes involved in Moco biosynthesis [22]. So far, the best characterized chaperones are FdhD from *E*. *coli* and FdsC from *R*. *capsulatus* [19–21].

Here, we addressed the questions whether FdsC from *R*. *capsulatus* and FdhD from *E*. *coli* are functional homologues and have exchangeable roles. Both chaperones are essential for their corresponding FDH enzyme(s), but so far, their exchangeable roles have not been investigated in detail. The crystal structure of dimeric *E*. *coli* FdhD in complex with GDP showed two symmetrical binding sites for nucleotides coming from the arm in the N-terminal domain of one monomer and from the C-terminal domain of the adjacent monomer (Fig 2) [19]. Based on the GDP binding sites the bis-MGD binding site was modeled on FdhD [19].

In this study, we compared the copurification of the chaperones with bound bis-MGD directly. We concluded that the bis-MGD cofactor bound to FdhD is present in a form with a higher saturation of the terminal sulfido-ligand. This was confirmed by a higher activity of TorA obtained after reconstitution with the cofactor bound to FdhD in comparison to FdsC. Recently it was shown that TorA contains a terminal sulfido ligand at the molybdenum atom that is essential for obtaining a fully active enzyme [35]. Thus, our conclusions seem feasible, since the higher activity was not based on a better transfer of the bis-MGD cofactor from FdhD to TorA.

We further investigated the roles of the cysteines in the conserved CXXC motif present in groups I and II of FdhD-like chaperones. This motif is not present or is modified in enzymes of group III (Fig 1). We showed that the cysteines did not impact the copurification of FdhD or FdsC with bis-MGD (Fig 3). However, both cysteines of FdhD had an impact on the activity of either *E*. *coli* (FdoGHI)_3_ or *R*. *capsulatus* (FdsGBA)_2_ (Fig 6). For *R*. *capsulatus* FdsC, in contrast, no difference in FDH activity was observed when the cysteine variants were used for the maturation of either *E*. *coli* (FdoGHI)_3_ or *R*. *capsulatus* (FdsGBA)_2_ (Fig 6B and reference [21]). Since no differences in bis-MGD transfer to a recipient protein was obtained between the FdhD-like chaperone and its cysteine variants (Fig 4), we investigated the interaction and sulfur transferase activity with IscS, the interaction partner protein from which the sulfur atom for bis-MGD sulfuration is obtained.

While FdhD wild-type was able to enhance the l-cysteine desulfurase activity of IscS, this effect was absent in the cysteine substitution variants (Fig 7) [20]. Further, this stimulating effect is an exclusive characteristic for FdhD with its partner protein IscS, since FdhD was unable to stimulate the l-cysteine desulfurase activity of the three l-cysteine desulfurases present in *R*. *capsulatus*. Moreover, the *R*. *capsulatus* FdsC chaperone does not provide this stimulating effect neither with the l-cysteine desulfurases from its own organism nor with the IscS protein from *E*. *coli*. Studies on the interactions between FdhD and FdsC and the different l-cysteine desulfurases revealed that FdhD and IscS form tight complex that can be copurified (Fig 8). Cys121 in *E*. *coli* FdhD seems to have an additional exceptional role in enhancing the l-cysteine desulfurase activity exclusively of IscS. This role might be especially required for the *E*. *coli* IscS protein, for which numerous interaction partners have been identified. IscS was additionally shown to interact with a number of acceptor proteins for delivery of sulfur including the involvement of *(i)* IscU, CyaY, Fdx and IscX for Fe-S cluster formation, *(ii)* TusA for either the (c)mnm^5^s^2^U34 modifications of tRNA or the biosynthesis of Moco, and *(iii)* ThiI for the synthesis of thiamine or the s^4^U8 modification of tRNA [36, 37]. Different binding sites for some of these molecules were mapped [38], ensuring either the simultaneous binding or a competitive binding on overlapping binding sites. While the binding site of FdhD on IscS has not been mapped so far, the stimulating effect on the activity of IscS might ensure that the sulfur is shuttled in the direction of bis-MGD sulfuration after binding of FdhD. It is possible that the stimulation of the l-cysteine desulfurase activity of IscS is triggered by a specific interaction with the disordered loop at the active site of IscS [39]. This has to be investigated in detail in future studies, *i*.*e*. by solving the co-crystal structure of IscS with FdhD. The reason why other FdhD-like chaperones do not comprise the enhancing effect on the l-cysteine desulfurase activity might be based on differences in the interaction site. As shown for *R*. *capsulatus* FdsC in this study, the interaction between both enzymes is weaker, since protein complexes were not copurified (Fog 8). However, since the bis-MGD cofactor on FdsC was identified to be present in its sulfureted form, *E*. *coli* IscS is able to deliver the sulfur to FDsC. Since in *R*. *capsulatus* three l-cysteine desulfurases are present which in total might have a lower number of interacting proteins [33], a more transient interaction might be sufficient to shuttle the sulfur directly in the direction of bis-MGD sulfuration. Since the specific roles of the three l-cysteine desulfurases have not been characterized in *R*. *capsulatus* so far, this assumption is of course speculative. In the organisms in which the CXXC motif is completely absent in the FdhD-like chaperone, the sulfur transfer pathway and the involvement of the corresponding l-cysteine desulfurase might differ. In organisms like *Bacillus subtilis*, numerous l-cysteine desulfurases were identified with a specific role for the synthesis of one sulfur-containing molecule [40]. Since these proteins are consequently not involved for sulfur transfer to several acceptor proteins and are specific for one particular pathway [41], the mode of interaction and the involvement of an additional cysteine on FdhD might serve a different role. Thus, for each organism the interaction of the FdhD-like chaperone with its partner l-cysteine desulfurase needs to be investigated in detail. Unfortunately, so far in many organisms the FdhD-like enzyme, the partner FDH enzymes and the corresponding l-cysteine desulfurases are largely uncharacterized.

**Fig 9:**
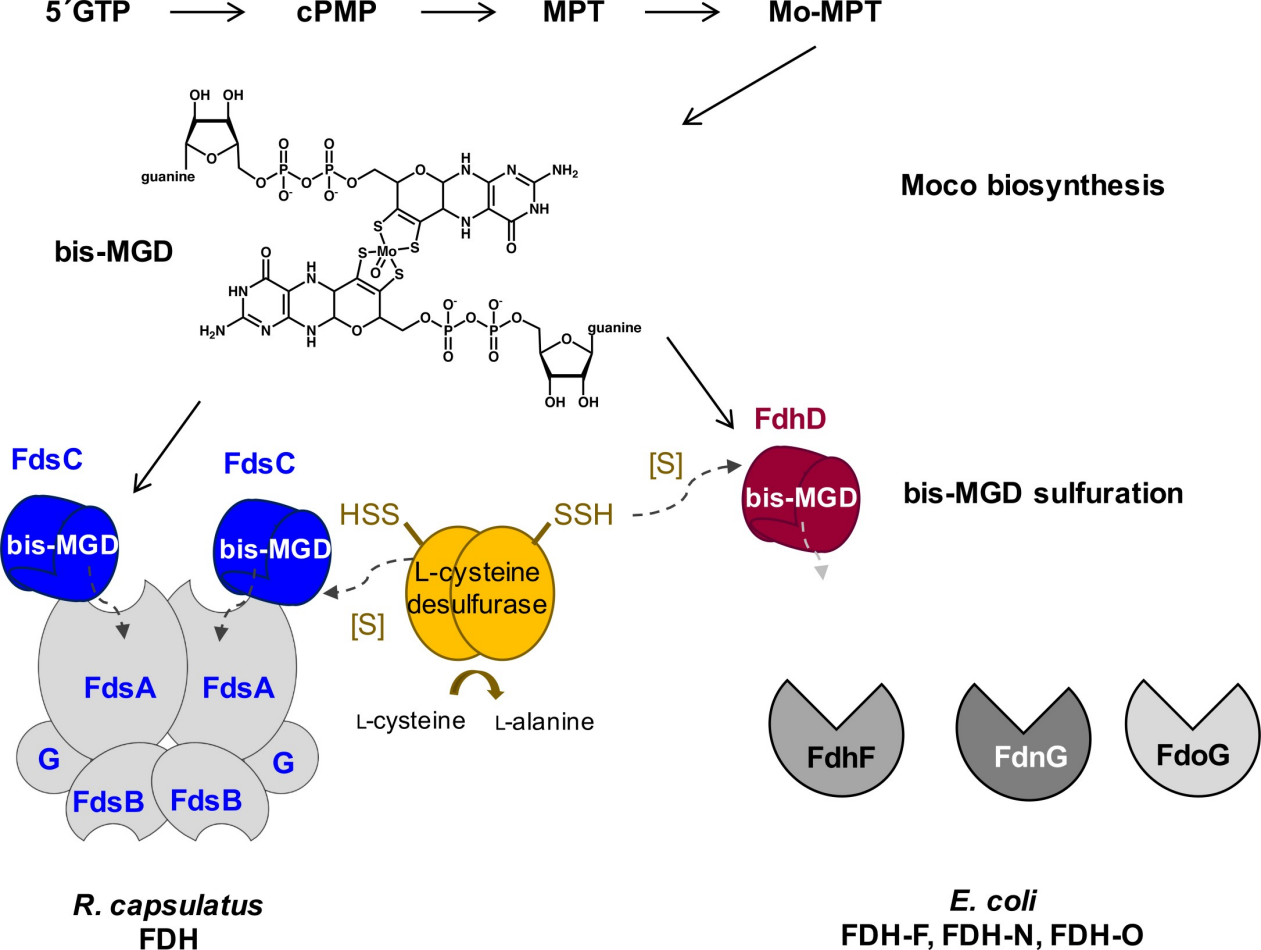
Model for bis-MGD sulfuration and insertion into the target enzymes by involvement of FdsC and FdhD. FdsC and FdhC bind the bis-MGD cofactor synthesized by the Moco biosynthesis machinery and provide the sulfurated bis-MGD for the FDHs of *R*. *capsulatus* and *E*. *coli*, respectively. Both chaperones are involved in the sulfuration of bis-MGD. FdhD interacts with the L-cysteine desulfurase IscS. The corresponding L-cysteine desulfurase for *R*. *capsulatus* has not been identified to date. FdsC and FdhD are functional homologues since the can replace each other in the maturation of the respective FDH.

In total, we conclude that the chaperones FdsC and FdhD have exchangeable roles. Both FdsC and FdhD can substitute each other in the maturation of the FDH partner enzyme(s) (Fig 9). This was not expected, since it has been suggested before that the system-specific chaperone coevolved with its corresponding partner molybdoenzyme. *E*. *coli* FdhD was shown before to be the chaperone for all three *E*. *coli* FDH enzymes, FdhF, (FdnGHI)_3_ and (FdoGHI)_3_ [20]. The crystal structures of FdhF and (FdnGHI)_3_ were solved and revealed to be highly identical [28, 42]. The crystal structure of *R*. *capsulatus* (FdsGBA)_2_ is not available so far. From amino acid sequence alignments, however, it becomes clear that the bis-MGD binding domain of FdsA is highly conserved and might provide a similar fold as the *E*. *coli* enzymes. Due to the high conservation of the bis-MGD containing domain of all FDH enzymes both FdhD and FdsC are expected to bind to a common motif present in all FDH enzymes, which then provides the basis of the exchangeable roles of both chaperones. The specific binding site of the FdhD-like chaperone on its FDH target enzyme in addition to the mechanism of bis-MGD insertion needs to be clarified in more detail in future studies.

## Materials and methods

### Bacterial strains, plasmids, media and growth conditions

Bacterial strains, plasmids and primers are listed in Table 1. *E*. *coli* BW25113 *ΔfdhD* cells were obtained from the Keio collection [43]. Cells were grown at 30°C under anaerobic conditions in LB medium containing 1 mM molybdate, 20 **μ**M isopropyl β-D-1-thiogalactopyranoside (IPTG), 150 **μ**g/mL ampicillin for 24 h. For site-directed mutagenesis and construction of the FdhD variants C121A, C124A and C121A/C124A, the expression vector pNB10 [21] was used as a template and base-pair exchanges were introduced by polymerase chain reaction mutagenesis. In general, to guarantee comparable growth conditions for the *E*. *coli* and *R*. *capsulatus* used, and protein expression was generally carried out at 30°C.

**Table 1:** Bacterial strains and plasmids used in this study.

	genotype/characterization	refs
**plasmids**		
pNBfdsC04	*fdsC*^C104A^ gene cloned into *Nco*I/*Sac*I site of pTrcHis, Amp^R^	[21]
pNBfdsC05	*fdsC*^C107A^ gene cloned into *Nco*I/*Sac*I site of pTrcHis, Amp^R^	[21]
pNBfdsC06	*fdsC*^C104A/C107A^ gene cloned into *Nco*I/*Sac*I site of pTrcHis, Amp^R^	[21]
pNBfdsC07	*fdsC*^C104A^ gene cloned into *Nde*I/*Sac*I site of pTrcHis, expressing N-terminally tagged His_6-_FdsC^C104A^, Amp^R^	this work
pNBfdsC08	*fdsC*^C107A^ gene cloned into *Nde*I/*Sac*I site of pTrcHis, expressing N-terminally tagged His_6-_FdsC^C107A^, Amp^R^	this work
pNBfdsC09	*fdsC*^C104A/C107A^ gene cloned into *Nde*I/*Sac*I site of pTrcHis, expressing N-terminally tagged His_6-_FdsC^C104A/C107A^, Amp^R^	this work
pNB10	*fdhD* gene cloned into *Nde*I/*Sal*I site of pTrcHis, expressing N-terminally tagged His_6_-FdhD, Amp^R^	this work
pNB11	*fdhD*^C121A^ gene cloned into *Nde*I/*Sal*I site of pTrcHis, expressing N-terminally tagged His_6_-FdhD^C121A^, Amp^R^	this work
pNB12	*fdhD*^C124A^ gene cloned into *Nde*I/*Sal*I site of pTrcHis, expressing N-terminally tagged His_6_-FdhD^C124A^, Amp^R^	this work
pNB13	*fdhD*^C121A/C124A^ gene cloned into *Nde*I/*Sal*I site of pTrcHis, expressing N-terminally tagged His_6_-FdhD^C121A/C121A^, Amp^R^	this work
pNB14	*fdhD* gene cloned into *Nco*I/*Sal*I site of pTrcHis, Amp^R^	this work
pNB15	*fdhD*^C121A^ gene cloned into *Nco*I/*Sal*I site of pTrcHis, Amp^R^	this work
pNB16	*fdhD*^C124A^ gene cloned into *Nco*I/*Sal*I site of pTrcHis, Amp^R^	this work
pNB17	*fdhD*^C121A/C124A^ gene cloned into *Nco*I/*Sal*I site of pTrcHis, Amp^R^	this work
pMN20	*nifS4* gene cloned into *Nde*I/*Xho*I site of pET28a, Km^R^	[33]
pMN51	*nifS3* gene cloned into *Nhe*I/*Xho*I site of pET28a, Km^R^	[33]
pMN54	*NifS2Δ1–188* gene cloned into *Nde*I/*Sal*I site of pET28a, Km^R^	[33]
pSL209	*iscS* gene cloned into *Nco*I/*Bam*HI sites of pET15b, Amp^R^	[54]
pTHfds02	*fdsC* gene cloned into *Nhe*I/*Sac*I site of pTrcHis, expressing N-terminally tagged His_6-_FdsC, Amp^R^	[30]
pTHfds14	*fdsC* gene cloned into *NcoI*/*Sac*I site of pTrcHis, Amp^R^	this work
pTHfds15	*fdsGBA* genes cloned into *SacI-/SalI* site (MSCI) and *fdsD* gene cloned into *NdeI/XhoI* (MSCII) site of pACYC-duet1, co-expressing N-terminally tagged His_6_-FdsGBA and FdsD, Cm^R^	[21]
pTorA	*torA* gene cloned into *Xba*I/*Hind*III site of JF119EH, Amp^R^	[55]
**strains**		
*ΔfdhD* (DE3)	JW3866-3, *Δ(araD-araB)567*, *ΔlacZ4787(*::*rrnB-3)*, *λ-*, *rph-1*, *ΔfdhD758*::*kan*, *Δ(rhaD-rhaB)568*, *hsdR514 λ(DE3)*	[43]
*ΔfdoG*	JW3865-2, *F-*, *Δ(araD-araB)567*, *ΔlacZ4787(*::*rrnB-3)*, *λ-*, *rph-1*, *ΔfdoG757*::*kan*, *Δ(rhaD-rhaB)568*, *hsdR514*	[43]
BL21 (DE3)	ompT gal dcm lon hsdSB(rB- mB-) λ(DE3)	Novagen
RK5200	F- araD139*Δ*(argF lac)U169 deoC1 flbB5201 gyrA219 relA1 rpsL150 non-9 ptsF25 chlA200::Mucts *Δ*moaA::Km	[56]

### Expression and purification of recombinant proteins

FdsC, its variants (from plasmids pTHfds02, pNBfds07, pNBfds08, pNBfds09, Table 1), FdhD and its variants (from Plasmids pNB10, pNB11, pNB12, pNB13, Table 1) were purified as described before for FdsC [21]. For co-expression of *R*. *capsulatus* FDH (FdsGBA + FdsD) with FdsC, FdhD or its cysteine variants, *E*. *coli* BW25113 *ΔfdhD* cells were transformed with pTHfds15 and either pTHfds14 pNB14, pNB15 pNBfds16 or pNBfds17. *R*. *capsulatus* (FdsGBA)_2_ was expressed and purified as described previously [30]. Apo-TorA was purified from soluble extract of RK5200 strain transformed with pTorA grown at 30°C 130 rpm by Ni-NTA chromatography as described previously [44, 45]. Expression and purification of IscS (pSL209), NifS2Δ1–188 (pNM54), NifS3 (pNM51), and NifS4 (pNM20) were performed as described previously [33, 46].

### Interaction study by size exclusion chromatography

20 **μ**M IscS was mixed with 40 **μ**M FdhD or FdsC and incubated in 100 mM potassium phosphate buffer, pH 8.0 for 30 min at room temperature. The proteins were loaded onto a Superdex 200 column equilibrated in the same buffer. Proteins were separated at a flow rate of 1 mL/min, and the elution profile was recorded at 280 nm. The proteins in the elution fractions were separated by 15% SDS-PAGE.

### Cofactor analysis

200 **μ**M of purified chaperone (FdsC, FdsC-C104A, FdsC-C107A, FdsC-C104A/C107A, FdhD, FdhD-C121A, FdhD-C124A, FdhD-C121A/C124A) or 2 **μ**M reconstituted TMAO reductase were incubated in acidic I_2_/KI-HCl at room temperature to release and oxidize bis-MGD to its fluorescent degradation product FormA-GMP. Form A-GMP was separated by a C18 reversed-phase high pressure liquid chromatography column (4.6 x 250-mm Hypersil ODS, 5-**μ**m particle size) after the method described previously [47]. Metal analysis was performed using PerkinElmer Life Sciences Optima 2100DV inductively coupled plasma optical emission spectrometer as described earlier [33].

### *In vitro* reconstitution of apo-TorA

8 **μ**M apo-TorA was incubated with 50 **μ**M FdsC, FdsC-C104A, FdsC-C107A, FdsC-C104A/C107A, FdhD, FdhD-C121A, FdhD-C124A, or FdhD-C121A/C124A in 100 mM potassium phosphate buffer for 2 hours at room temperature under anaerobic conditions. TMAO reductase activity was measured under anaerobic condition following the oxidation of reduced benzyl viologen in the presence of 0.1 **μ**M TMAO (ε_NADH_ = 7,400 M^-1^cm^-1^) as described by Kaufmann et al. [35]. For detailed analysis of reconstituted TMAO-reductase with FdsC and FdhD, TorA was incubated with FdsC or FdhD for 7 hours at 37°C and purified from the mixtures by gel filtration on a Superdex 200 10/300GL column (GE Healthcare). The containing TorA were combined and used for cofactor analysis and activity measurements.

### Enzyme assays

*R*. *capsulatus* (FdsGBA)_2_ activity was measured with 120–300 nM enzyme in the assay by recording the production of NADH at 340 nm (ε_NADH_ = 6,220 M^-1^cm^-1^) as described previously [30].

l-cysteine desulfurase activity of IscS, NifS2Δ1–188, NifS3, and NifS4 was measured by determination of the rate of sulfide production as described previously [33, 48]. 1 **μ**M l-cysteine desulfurase was mixed in a 1:2 ratio with FdsC, FdhD, and SufE in a total volume of 480 **μ**l containing 100 mM potassium phosphate, 200 mM NaCl, 10 **μ**M pyridoxal phosphate, and 1 mM dithiothreitol, pH 8.0. After incubation for 10 min at 30°C, the reactions were stopped by the addition of 60 **μ**l of 20 mM N,N-dimethyl-p-phenylenediamine in 7.2 M HCl and 60 **μ**l of 30 mM FeCl_3_ in 1.2 M HCl. After additional incubation for 20 min at 30°C, precipitated protein was removed by centrifugation, and methylene blue was measured at 670 nm. A standard curve was generated using sodium sulfide in a range of 0–25 **μ**M.

### In-gel staining of FdhO activity

For detection of FdhO activity, BW25113 cells, *ΔfdhO* cells, *ΔfdhD* cells and *ΔfdhD* cells complemented with plasmids coding for FdhD and FdsC as well as for their cysteine variants (pNB14, pNB15, pNB16, pNB17, pTHfds14, pNBfds04, pNBfds05, pNBfds06) were grown anaerobically in 55 ml LB medium supplemented with 10 **μ**M sodium molybdate, 0.2% sodium formate and 20 **μ**M IPTG for 16 hours (starting with OD_600_ of 0.05). After harvesting cells were resuspended in 1.5 ml 50 mM potassium phosphate buffer, pH 6.8 containing 1% (v/v) Triton X-100 and were disrupted by sonication. Cell debris were removed by centrifugation and the protein concentration of the supernatant was measured by the BCA method as described by the manufacturer (Micro BCA Protein Assay Reagent Kit, Pierce). 40–60 **μ**g of total proteins were separated by non-denaturing PAGE using Tris-Glycin with 0.1% (v/v) as running buffer. Detection of FdhO activity was performed as described using 5 mM sodium formate as substrate, 1 mM nitroblue tetrazolium as electron acceptor and 0.5 mM phenazine methosulfate as mediator in 50 mM potassium phosphate, pH 6.8 [49]. The gels were incubated in staining solution for 30 min at 37°C under aerobic conditions.

### Phylogenetic data analysis

Amino acid sequences of 41 FdhD-like sequences from various taxa (bacteria: actinobacteria, firmicutes, cyanobacteria and proteobacteria, and archaea, Table 2) were extracted from ENSEMBLE and amino acid sequence alignments were created with Muscle [50]. The FdhD/FdsC amino acid sequences were selected from different organisms including characterized and uncharacterized FDH proteins to have a broad selection of sequences which was supposed to enable the analysis whether a splitting in the different groups of FDHs occurs [22]. The tree was constructed using the Maximum Likelihood method based on the Dayhoff matrix based model [51]The tree is drawn to scale, with branch lengths in the same units as those of the evolutionary distances used to infer the phylogenetic tree. The evolutionary distances were computed using the Dayhoff matrix based method and are in the units of the number of amino acid substitutions per site [52]. The scale bar indicates 0.2 substitutions per site. Numbers near branches indicate the bootstrap proportion for 100 replica using the same method. All positions containing gaps and missing data were eliminated. There was a total of 174 positions in the final dataset. Evolutionary analyses were conducted with the software MEGA7 [53].

**Table 2:** Protein accession numbers used for the phylogenetic tree. NCBI protein accession numbers for each FdhD/fdsC amino acid sequence in alphabetical order of the organisms.

Accession Number	Organism
ABR74626	*Actinobacillus succinogenes*
KAJ33787	*Agrobacterium tumefaciens*
ELT44815	*Arthrobacter nitrophenolicus*
ACO76640	*Azotobacter vinelandii*
CAB15688	*Bacillus subtilis*
KHS53770	*Brevibacterium linens*
AFS79899	*Clostridium acidurici 9a*
CAF19235	*Corynebacterium glutamicum*
SCV00736	*Cupravidus necator*
AGW12659	*Desulfovibrio gigas*
YP_009800	*Desulfovibrio vulgaris str*. *Hildenborough*
AIZ53701	*Escherichia coli K13*
BAD74746	*Geobacillus kaustophilus*
ACY12706	*Haliangium ochraceum*
ADJ24264	*Hyphomicrobium denitrificans*
AEB94994	*Metallosphaera cuprina*
AIS30994	*Methanobacterium formicicum*
ABQ86500	*Methanobrevibacter smithii*
ABQ87597	*Methanobrevibacter smithii*
WP_048064104	*Methanococcus maripaludis*
ABR54454	*Methanococcus vannielii SB*
ACK84408	*Methylobacterium extorquens CM5*
ACT50427	*Methylovorus glucosetrophus*
CDO44190	*Mycobacterium bovis*
ACV78244	*Nakamurella multipartita*
ACC79387	*Nostoc punctiforme*
BAF60791	*Pelotomaculum thermopropionicum*
ABG41355	*Pseudoalteromonas atlantica*
ABR85479	*Pseudomonas aeruginosa*
ETD87413	*Rhodobacter capsulatus*
OPF94773	*Rhodopseudomonas palustris*
ABI70605	*Shewanella frigidimarina*
AAP18794	*Shigella flexneri*
ADD40018	*Stackebrandtia nassauensis*
CAI81841	*Staphylococcus aureus*
BAG17950	*Streptomyces griseus*
BAB65040	*Sulfolobus tokodaii*
ABK15737	*Syntrophobacter fumaroxidans MPOB*
YP_845333.1	*Syntrophobacter fumaroxidans MPOB*
ACP05777	*Vibrio cholerae*
WP_011137999	*Wolinella succinogenes*
